# Immunogenicity of CAR-T Cell Therapeutics: Evidence, Mechanism and Mitigation

**DOI:** 10.3389/fimmu.2022.886546

**Published:** 2022-05-23

**Authors:** Aalia N. Khan, Ambalika Chowdhury, Atharva Karulkar, Ankesh Kumar Jaiswal, Ankit Banik, Sweety Asija, Rahul Purwar

**Affiliations:** Department of Biosciences and Bioengineering, Indian Institute of Technology Bombay, Mumbai, India

**Keywords:** chimeric antigen receptor, monoclonal antibodies, scFv, cellular immunity, anti-CAR antibodies, persistence, immunogenicity

## Abstract

Chimeric antigen receptor T cell (CAR-T) therapy demonstrated remarkable success in long-term remission of cancers and other autoimmune diseases. Currently, six products (Kymriah, Yescarta, Tecartus, Breyanzi, Abecma, and Carvykti) are approved by the US-FDA for treatment of a few hematological malignancies. All the six products are autologous CAR-T cell therapies, where delivery of CAR, which comprises of scFv (single-chain variable fragment) derived from monoclonal antibodies for tumor target antigen recognition is through a lentiviral vector. Although available CAR-T therapies yielded impressive response rates in a large number of patients in comparison to conventional treatment strategies, there are potential challenges in the field which limit their efficacy. One of the major challenges is the induction of humoral and/or cellular immune response in patients elicited due to scFv domain of CAR construct, which is of non-human origin in majority of the commercially available products. Generation of anti-CAR antibodies may lead to the clearance of the therapeutic CAR-T cells, increasing the likelihood of tumor relapse and lower the CAR-T cells efficacy upon reinfusion. These immune responses influence CAR-T cell expansion and persistence, that might affect the overall clinical response. In this review, we will discuss the impact of immunogenicity of the CAR transgene on treatment outcomes. Finally, this review will highlight the mitigation strategies to limit the immunogenic potential of CARs and improve the therapeutic outcome.

## Introduction

Cancer treatment advanced dramatically in recent years, however, cancer still continues to be a global challenge due to poor response rate of available therapies in long-term remission (1, 2). Despite an initial curative response, cancer patients develop resistance and eventually relapse in response to existing conventional therapies (3, 4). Due to the complexity of tumorigenesis, finding a promising immunotherapy that targets tumors at both the cellular and genetic levels is extremely crucial (5). Chimeric Antigen Receptor T (CAR-T) cell therapy is a type of adoptive immunotherapy that has emerged as novel therapeutics in which immune cells are genetically engineered to target tumor-associated antigens, preventing them from evading immune responses (6). In the 1980s, Esshar successfully demonstrated the principle of genetically redirecting Cytotoxic T-lymphocytes to tumor cells, suggesting their anti-tumor potential against human tumors (7). Since then, CAR-T cell therapy has emerged as a revolutionary new pillar in cancer care.

A Chimeric antigen receptor (CAR) are fusion of proteins which redirect T cell specificity towards surface molecules expressed on tumor cells independently of the conventional T cell receptor (TCR)–major histocompatibility complex (MHC) interactions (8, 9). As a result, these CARs circumvent some of the drawbacks of designed TCRs, such as the requirement for MHC identification and presentation of the target cancer antigen. CAR construct primarily comprises of four domains, the ectodomain for specific target antigen recognition and endodomain that provides costimulatory and activation signals. These two domains are linked by the hinge and transmembrane domains, which also influence their functional characteristics. The antigen-recognition domain is typically a mouse-derived monoclonal antibody in the form of a continuous peptide single-chain variable fragment (scFv) directed through a flexible extracellular spacer domain. The extracellular region is composed of these scFv directed towards a cell surface antigen on tumor cell, while the intracellular domain is made up of fused signaling domains from a natural TCR complex as well as costimulatory components (10–13).

CAR-T cells are manufactured *ex vivo* by modifying T cells derived from the patient’s peripheral blood. CAR genes are delivered into T cell genomes using a wide range of gene transfer techniques involving viral or non-viral vectors (14). One of the most widely used gene transfer techniques is T cell transduction with replication-deficient lentiviral vectors that integrate the CAR expression cassette into the T cell genome. Furthermore, these CAR-T cells are multiplied to a large number until a dosage is achieved. After infusion into patients, these cells can then detect and eliminate tumor cells expressing the target antigen (15–17). Both autologous and allogeneic CAR-T cells have successfully progressed from preclinical to clinical development; however, only autologous CAR-T cells are currently approved to treat cancer patients (18, 19).

More than 500 CAR-T cell therapies have undergone clinical trial worldwide. Second generation patient derived CAR-T cells have demonstrated significant clinical responses and durable remissions in B-cell hematologic malignancies, leading to the approval of CAR-T therapy to treat patients with cancer (20). There are currently six FDA approved CAR-T cell therapies, all against hematologic malignancies. Tisagenlecleucel, axicabtagene ciloleucel, brexucabtagene autoleucel and lisocabtagene maraleucel are CD19-targeted CAR-T cell products, being used to treat relapsed or refractory B cell acute lymphoblastic leukemia (r/r B-ALL), and non-Hodgkin’s lymphomas (r/r NHLs) (21–24). Patients with B-ALL or some aggressive NHL have shown that CD19-targeted CAR-T cells can induce full remissions in a subset of intensively pre-treated patients with extensive disease. In March 2021, Idecabtagene vicleucel, the first ever non-CD19 CAR-T cell therapy was approved for the treatment of multiple myeloma (25). Ciltacabtagene autoleucel is the most recent B-cell maturation antigen (BCMA) directed CAR-T cell therapy approved for adults with relapsed and/or refractory multiple myeloma (r/r MM) (26).

Despite the remarkable success of CAR-T cell therapy, it has been associated with side effects which limit its benefits. Balancing CAR-T cell efficacy, and safety has proven to be a major challenge. CAR-T cell engagement with the tumor cells has been known to lead to life threatening adverse events including early onset events such as cytokine release syndrome (CRS), and neurotoxicity and late toxicities such as Hemophagocytic lymphohistiocytosis (HLH). CRS is caused due to rapid immune activation induced by CAR-Ts, leading to considerable elevation in the levels of inflammatory cytokines. It is the most common acute toxicity associated with CAR-T therapy. CRS initially manifests with fever and hypotension, but may progress to life-threatening vasodilatory shock, capillary leak, hypoxia, and end-organ dysfunction (27, 28). Neurotoxicity, recently recognized as ICANS (Immune effector Cell–Associated Neurotoxicity Syndrome) has been reported in patients, manifesting as encephalopathy, confusional state, aphasia, myoclonus and other central nervous system disorders (29).

Another obstacle to CAR-T cell efficacy, is the immunogenicity of CAR-T cells. The various components of the CAR expressed on the T cell surface may be identified as foreign and elicit an immune response. All CAR-T therapies approved for use today, except for ciltacabtagene autoleucel, utilize murine scFv. Ciltacabtagene autoleucel is a dual epitope-binding CAR-T cell therapy directed against BCMA composed of llama-derived single-domain heavy-chain antibodies (30). The presence of such non-human sequences in CAR construct has been seen to lead to induction of immune responses including T-cell mediated immunity and generation of anti-CAR antibodies. Few evidence suggests that such immune activation may result in the premature clearance of CAR-T cells, as well as hinder repeat dosing (31–33).

In this review, we present the clinical evidence of immune responses elicited by CAR-T cells, and the impact of such immunogenicity on CAR-T cell efficacy as well as patient safety. Finally, we discuss the methods being explored to reduce CAR-T cell immunogenicity and highlight the importance of the treatment regime in managing immunogenicity.

## Clinical Evidence of CAR Immunogenicity

**Figure 1:**
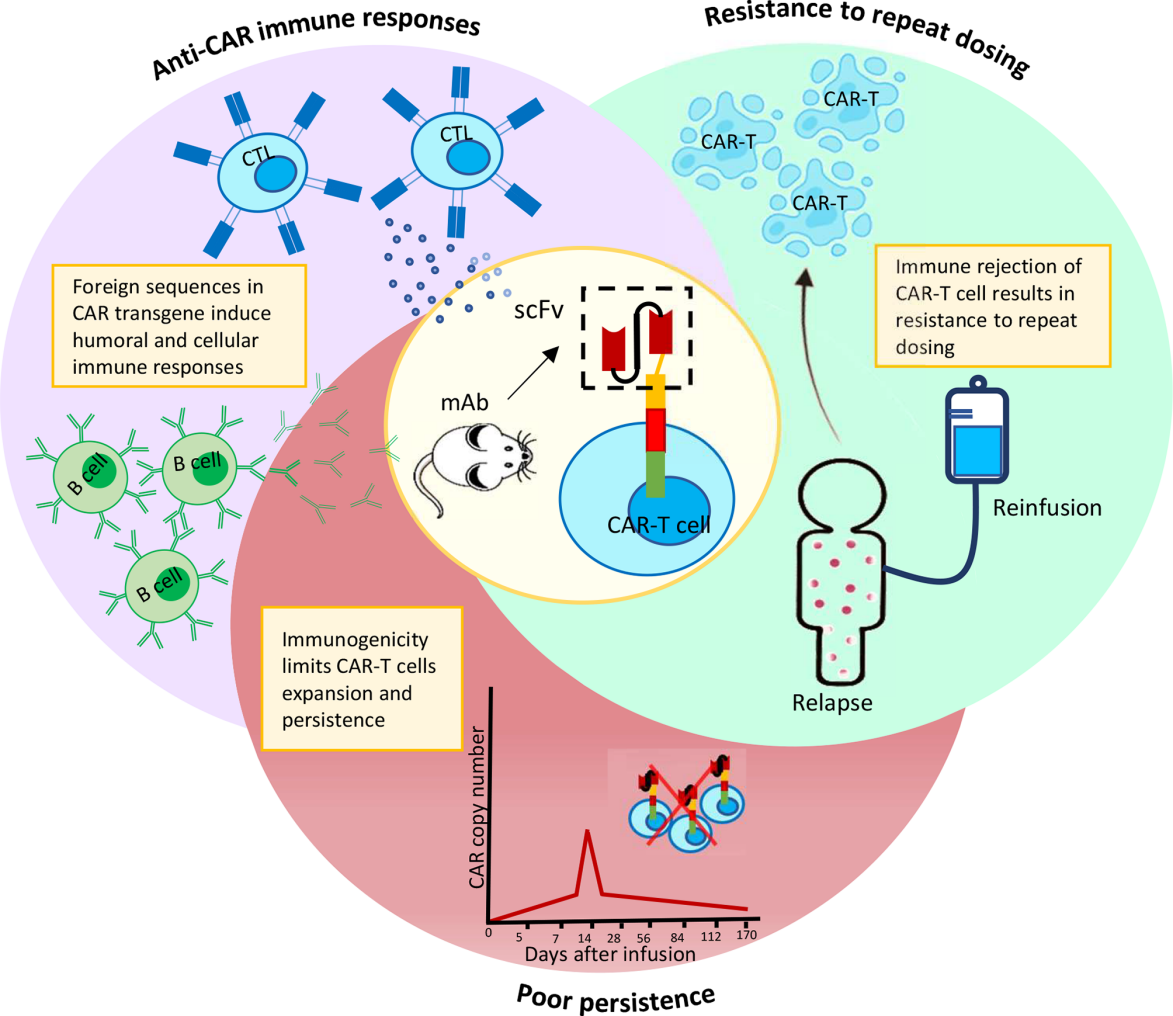
Immuno-activation by murine scFv results in CAR-T cells elimination prematurely and resistance to repeat dosing.

A recent study discovered CAR+ cells in the bloodstream of two chronic lymphocytic leukemia (CLL) patients who were infused with CT019, indicating a decade-long sustained remission (34). This study highlights, the persistence of CAR-T cells in circulation to be critical for a successful remission upon CAR-T treatment. Structural components of a CAR can easily affect such long-term persistence of CAR-T cells (35, 36). Inclusion of non-human sequences in the CAR construct, particularly scFvs, have been linked to anti-CAR humoral or cellular immunity, resulting in rapid clearance of CAR-T cells from circulation **(** **Figure 1****)**. Other CAR-T components such as the suicide domain, as well as the presence of residual viral proteins used in the gene editing step of CAR-T manufacturing, are few other risk factors for immunogenicity induction (37). In this section, we discuss about the various types of immune responses against a range of CAR-T cells and their impact on overall clinical outcome.

### Non-Human Sequences in scFv Lead to Development of Anti-CAR Antibodies, Limiting Repeat Dosing and Therapeutic Outcome

The development of anti-CAR antibodies is one of the major concerns which could potentially lead to treatment failure. In such circumstances, the patient’s need for repeat infusion becomes important. However, due to the ability of CAR-T cell products to induce host immunological responses, patients may develop resistance to repeat doses. A study by Turtle et al., demonstrated that B-ALL patients who relapsed after CD19 CAR-T cell therapy did not respond to repeat dose of CD19 CAR-T cells (38). In another study, Maus et al., found that multiple repeat doses of murine mesothelin CAR-T cells caused allergic reactions in a patient, which resulted in immediate death due to cardiac arrest (39).

Some of the studies have reported the development of a humoral response against the CAR-transgene. In a recent study conducted by Xu et al., two camel-derived antigen-binding domains were fused with a single CAR construct to generate a bispecific CAR-targeting two distinct BCMA epitopes. In patients with multiple myeloma, this CAR exhibited remarkable efficacy, with the highest reported complete remission (CR) rate of nearly 76% (13/17) (NCT03090659). However, antibodies against the CAR were reported in all patients who relapsed after a complete remission and were linked to a drop in the number of circulating CAR-T cells (40).

In an approach described by Lamers et al., a humoral anti-idiotypic and neutralizing response was reported for an autologous CAR-T cell therapy specific to the carbonic anhydrase IX (CAIX) in metastatic renal cell carcinoma (RCC) patients (DDHK97-29/P00.0040C) (41). The scFv of CAIX CAR-T cells was derived from murine monoclonal antibody G250 (G250 mAb) which recognizes an epitope on CAIX that is frequently overexpressed on the surface of RCC. The study included 11 subjects, 3 of whom received an additional pre-treatment of low dose parental cG50 antibody with a strategy to block CAIX CAR recognition of cognate antigen on normal liver tissue while still leaving accessible CAIX at RCC tumor sites. The remaining 8 patients received no pre-conditioning before CAR-T infusion. Human antibody responses to chimeric G250 (HACA) were analyzed using sandwich ELISA to monitor anti-CAR immune response in patients treated with CAIX CAR-T cells. After the start of the treatment, 6 of the 11 patient sera tested positive for HACA, with the reactivity of HACA being directed against the idiotype (Id) of scFv (cG250 idiotype). In a cytotoxicity assay, HACA containing patient sera successfully blocked CAIX-CAR–mediated cytolysis, suggesting the anti-idiotype (anti-Id) CAR nature of the HACA, which limits the functional persistence of CAR-T cells. Surprisingly, no HACA was induced in patients who received an infusion of cG250 mAb prior to the CAR-T cells. Overall, the findings explained that humoral immune responses like HACA limit the functional peripheral persistence of CAR-T cells, and that pre-conditioning with appropriate immunosuppressive regimens can be beneficial in ameliorating the humoral immunogenicity profile of CARs (42).

Another study led by Hege et al., explained the outcomes from the first two clinical trials examining CAR-T cells in patients with solid tumors, with a focus on persistence and immunogenicity. This study described the induction of a humoral response towards the CAR-T72 construct, which targets the tumor associated glycoprotein (TAG-72). The construct was developed by humanizing TAG-72 specific antibody of murine origin (CC49) to treat metastatic colorectal cancer (CRC). Despite repeated high-dose CAR-T cell infusions (1×10^10^) in the majority of patients, the immune response led to the clearance of the infused TAG-72 CAR-T cells in <14 weeks (43). Blanco et al., also reported an increase in CAR-T cell clearance following the second and third infusions in some patients after exposure to murine version of CC49 antibody reactive with TAG-72. After being treated with CC49 mAb (n=33), about 70% of the patients experienced a human anti-mouse antibody (HAMA) response, while 54% of the patients exhibited an anti-idiotypic immune response. The findings from the above two studies suggests that murine residues in the humanized CC49 domain of CAR-T72 may have immunogenic potential, which could have resulted in immune rejection and resistance to subsequent doses. This aligned well with the observation that the original murine CC49 was highly immunogenic after a single dose, with a significant number of patients developing anti-idiotypic or HAMA immune responses (44).

Brian G. Till and colleagues administered anti-CD20 CAR-T cells to treat indolent B-cell lymphoma or Mantle cell lymphoma (NCT00012207). The scFv domain of the anti-CD20 CAR-T cell used in the investigation was derived from a murine antibody (Leu-16). As a result, assays were conducted to see if these murine-derived scFv could elicit an immunological response. Initially, there was no HAMA response observed in the patients. CAR-T cells were also detectable in the bloodstream for roughly 9 weeks post infusion. However, after 3- and 12-months following infusion, 2 out of 7 patients were found to be seropositive for HAMA (45). Anti-CAR antibody response was also reported in a study wherein the T cells were engineered to express α-folate receptor (FRα)-specific CAR domain based on a murine anti-FR antibody (MOv18). These FR-specific CAR-T cells were developed for treatment of metastatic ovarian cancer (NCT00019136). IFN-γ release was inhibited as a result of the antibody-mediated immune response against the CAR-T cells. It was even observed that the patient’s serum decreased CAR-T cytolytic potential against FR-expressing tumor cells post infusion. This suggested that anti-CAR antibodies may have significantly reduced the efficacy of CAR-T cells, resulting in their rapid clearance as seen in the trial (46). These cumulative clinical evidence suggest that humoral immunity may be linked to CAR-T cell inactivation, limiting the overall clinical outcome.

In contrast, immune responses elicited by CAR-T cells reported in few studies were not found to hamper the overall therapeutic function. Clinical investigations of the two FDA-approved tisagenlecleucel (KYMRIAH^®^) and axicabtagene ciloleucel (YESCARTA^®^) demonstrated that they hold the potential to elicit humoral responses (47, 48). Mueller and colleagues examined the pharmacological effects of tisagenlecleucel in r/r B-ALL patients (n=79, NCT02435849 and NCT02228096). Patients were tested for anti-murine CAR19 antibodies (mCAR19 Ab) before and after receiving tisagenlecleucel. 84.4% of tisagenlecleucel-treated study patients tested positive for mCAR19 Ab before infusion, while 36.7% developed induced humoral immunogenicity after infusion (49, 50). Awasthi et al., investigated similar immunogenicity profile of tisagenlecleucel in r/r DLBCL patients (n=111, NCT02445248). In this study, nearly 91.4% of patients exhibited pre-existing anti-mCAR19 Ab prior to infusion, while antibodies were found in only 5% of patients after infusion (51, 52). The presence of pre-existing antibodies in these patients was most likely caused by previous therapy with chimeric human/mouse monoclonal antibodies prior to CAR-T cell infusion. As a result, post treatment anti-mCAR19 Ab were considered positive only when the level was greater than the baseline level of the individual patient. On a side note, pre-existing or treatment-induced anti-mCAR19 Ab had no effect on efficacy, safety, or tumor relapse in both studies.

To evaluate immunogenicity profile of axicabtagene ciloleucel, a separate study was conducted for NHL. Patient were screened for the presence of binding antibodies specific to the murine-derived FMC63 using ELISA. Only 3 of the 94 subjects in the ZUMA-1 trial (NCT02348216) tested positive for murine antibodies prior to treatment; however, no anti-CAR response was observed in any of the patients after infusion. In ZUMA-5 study (n=148, NCT03105336), antibodies were detected in 13% of patients prior to infusion; however, 2% of patients who tested negative prior to treatment tested positive post treatment. Even in this study, the elicited immune response had no implication on efficacy, persistence, or safety (22, 53–55).

Despite the fact that both tisagenlecleucel and axicabtagene ciloleucel scFvs were derived from murine FMC63 and were identical, there was a significant difference in humoral immunogenicity induction when used for different indications. This suggests that the occurrence of an immune response to murine scFv, as well as the implications of such a response, are unclear, and that a thorough investigation is needed to better understand the role of immunogenicity in treatment outcomes.

### T Cell Immune Responses Directed Against Foreign Sequences in the CAR-Transgene Limit CAR-T Cell Persistence Impacting Clinical Outcome

Cellular immune responses against CAR-T cells likely originate from the processing and MHC dependent cross-presentation of foreign sequences. The CAR peptide (often comprising murine sequences) may be displayed by antigen presenting cells, leading to the priming of CAR-specific cytotoxic T cells (56). In some cases, the presence of CAR-specific cytolytic T cells have been associated with immune rejection of the adoptively transferred T cells, and subsequent treatment failure.

Multiple clinical trials have reported T cell immune responses against anti-CD19 CAR-Transgene components. Analysis of post infusion PBMCs has commonly been used to determine the presence of CAR-specific CD8^+^ T cell response. In 2015, a phase I dose escalation trial was conducted with FMC63-based anti-CD19 CAR-T cells to treat children and young adults with B-ALL (n=21, NCT01593696) (57). CAR-T cells could not be detected in any patient after day 68. Proliferation of T cells in post infusion samples was seen in response to autologous CAR-T cells, indicating a T cell-mediated clearance of CAR-T cells. Another trial conducted by Cameron J. Turtle also reported a similar lack of persistence (n = 29, NCT01865617) (38). Turtle and team conducted a phase I/II clinical trial in patients with refractory B-ALL in which CD8^+^ and CD4^+^ T cell subsets were separately modified to express a CD19-targeted CAR (FMC63-based) formulated in a defined ratio of CD4^+^:CD8^+^ CAR-T cells. Although an overall response of 50% was observed, a loss of CAR-T cells within 100 days was recorded in 9/10 patients, 8 of whom developed progressive disease. 5 patients with tumor progression or relapse were infused with a second dose of CAR-T cells, after which no expansion or proliferation of the CAR-T cells was seen, and no anti-tumor activity could be detected. In the treatment of follicular lymphoma by FMC63-derived CD19 CAR-T cells Michael C. Jensen et al., faced the same obstacle (n=5, NCT00182650) (58). The CAR-T cells failed to persist beyond a week even at higher doses. Further, a notable reduction was seen in the levels of CD19 CAR-T cells 24 hours after every additional infusion, as compared to the levels after the initial infusion.

Analysis of post infusion PBMCs has been a commonly used approach to determine the presence of CAR-specific CD8^+^ T cell response. In the trial led by Turtle, a ^51^Chromium release assay was performed to determine the presence of a CAR specific CD8^+^ T cell response, with pre and post infusion PBMCs that had been stimulated with autologous irradiated CAR-T cells (38). CAR-specific T cell responses was detected in all 5 patients, wherein CAR-T cells failed to persist after the second infusion. Jensen et al., assessed cellular immune responses against the therapeutic T cell product *ex vivo*, by TCR Vβ spectratyping, and CD107 degranulation assays (58). Analysis of the TCR Vβ gene revealed appearance of unique clonotypes in the post infusion PBMCs, which suggest the development of a new immunoreactive response. Flow cytometric analysis of post infusion PBMCs also showed significant surface expression of CD107, indicative of lysis associated degranulation. Such degranulation was not observed when pre-treatment PBMCs were analyzed, or when the PBMCs were co-cultured with control T cells. These results indicate the generation of an anti-transgene immune response mounted by the patients endogenous T cells.

Anti-CAR-Transgene responses have been noted in CAR-T cells targeting other tumors as well. In one such study, Lam et al., attempted to investigate if murine-derived scFv containing CARs could be immunogenic. A non-signaling CAR (11D5-3-NS) was generated in this work, with sequences that were identical to the CAR-BCMA that had previously been evaluated in clinical trials (NCT02215967). For two weeks, patient-derived PBMCs were continuously stimulated with 11D5-3-derived scFv expressing T-cells or 11D5-3-NS CAR-T cells. These stimulated PBMCs were subsequently co-cultured with autologous 11D5-3 expressing T-cells or negative control cells expressing NGFR, overnight. PBMC reactivity against 5/10 patients 11D5-3-CAR was detected using IFN-γ by ELISA. The levels of IFN-γ were about 3-4 times greater than the negative control, indicating an enhanced T-cell mediated immune response to murine-derived scFv (59, 60).

Cor H. J. Lamers and team targeted renal cell carcinoma with CAR-T cells directed towards Carbonic Anhydrase IX (CAIX) (61). No objective clinical response was reported in the study. In a majority of patients, the CAIX CAR-T cells showed poor peripheral persistence. Cellular anti-CAIX CAR-T cell immune responses were detected and evaluated as the underlying cause for the above phenomenon. No cell mediated immunity could be detected when fresh PBMCs collected pre, during or post treatment were simulated with transduced T cells. However, on 2-5 cycles of weekly coculture of the PBMCs with CAR-T cells, the anti-CAR-T effector population was amplified and detectable. A strong cellular immune response was noted in 7 out of 9 patients against CAR-T cells, but not against non-transduced T cells.

A step forward from the detection of T cell-mediated immune responses, multiple studies have utilized epitope mapping strategies to identify immunoreactive peptides within the CAR-transgene (38, 61). C.J. Turtle in his study stimulated post infusion T cells from a patient showing CAR-specific T cell response with pools of overlapping peptides from the CAR construct. An ELISpot assay was used to identify the peptide pools which induced IFN- γ secretion higher than that induced by T cells alone. Immunoreactive peptides which could bind to human Leukocyte antigen (HLA) molecules expressed by the CAR-T cells were detected in the complementarity–determining regions (CDRs) and framework region of the murine FMC63 scFv utilized in this study. In an independent study, Lamers incubated post treatment PBMCs with 11 amino acid overlapping 15-mer peptides spanning the complete CAIX-CAR protein. Responder PBMCs were tested for membrane expression of CD137. The samples testing positive were then tested using 19 matrix pools containing 8-10 peptides each and, finally with the identified candidate individual 15-mer peptides. HLA peptide binding predictions were carried out for the detected immunoreactive peptides. HLA binding, immune reactive epitopes were identified in the VH- (CDR) and the VK-framework region of the CAIX specific murine G250 scFv.

Anti-transgene cellular immune responses have also been noted against non-CAR elements (58). Patients with DLBCL were treated with autologous T cells expressing murine Leu 16 derived anti-CD20 CAR, along with a selection marker neoR, coding for neomycin phosphotransferase (n=2, BB-IND-8513/IRB 98142). T cell effector responses could be detected against the autologous CAR-T cells. This immune reactivity was found to be specific for the neomycin phosphotransferase. Induction of anti-transgene immune response was therefore seen to cause clearance of CAR-T cells especially on repeat dosing, which in some cases could be correlated with poor clinical outcome.

### Allogeneic CAR-T Cells and Immune Rejection

CAR-T cell therapy traditionally involves modification of individual patient’s T cells. While this approach allows for personalized therapy, the process of collection of patient cells and subsequent engineering is both time consuming and expensive. Further, there also exists the risk of manufacture failure due to the limited amount and poor quality of T cells obtained from the heavily pre-treated patients (62). To overcome these challenges, multiple research groups have attempted to create “off the shelf” or allogeneic CAR-T cells from the blood of healthy donors which would reduce the cost as well as the time required for CAR-T cell treatment.

The biggest challenge in developing such CAR-T cells is the genetic dissimilarity between the donor and recipient, leading to immunological incompatibility. The diverse range of non-self HLA molecules on the infused cells lead to Graft Versus Host Disease (GVHD). This has been known as one of the major limitations of allogeneic transplant therapies including CAR-T cell therapy (63). Another obstacle to developing allogeneic CAR-T cell therapy is the host derived immune response mediated by the patient’s alloreactive T cells (64). Even HLA matching does not completely eliminate the risk of alloreactivity (65).

To reduce the risks of GVHD and immune rejection, multiple groups have attempted to eliminate the expression of the endogenous TCR. Several gene editing techniques have been used to modify the CAR-T cells such as site-specific endonucleases, including transcription activator-like effector nucleases (TALENs), Mega-TALs, zinc-finger nucleases (ZFNs), and CRISPR/Cas9 (66–69). Other molecules targeted by gene editing include, β2-microglobulin, PD-1, and HLA-A2 and CD52 (70–72). Jacobson et al., developed PBCAR0191, an anti-CD19 allogeneic CAR-T cell, using site specific endonucleases to specifically insert a CD19 specific CAR into the T cell receptor alpha constant (TRAC) locus in cells harvested from healthy donors. In a phase I trial (NCT03666000), 13 patients were treated with PBCAR0191 with no reports of GVHD and comparable anti-tumor efficacy to autologous CAR-T cells (73).

Despite gene editing efforts, immune rejection of the allogeneic CAR-T cells remains a problem, limiting CAR-T cell expansion and persistence. Benjamin et al. tested allogeneic CD19 CAR-T cells in two phase I studies, treating pediatric (n = 7, NCT02808442 PALL study) or adult patients (n=14, NCT02746952 CALM study) with r/r B-ALL. The CD19 CAR-T cells used (UCAR19) in these studies were modified by TALENs to knock out the genes encoding the TCR α constant chain and CD52 (74). In 17 of the 21 patients, pre-infusion lymphodepletion was carried out with fludarabine (Flu), cyclophosphamide (Cy) and alemtuzumab, while 4 patients received only Flu and Cy. Flow cytometric analysis was carried out to detect host T cell levels after UCAR19 administration. Endogenous T cells were seen to recover faster in the patients not receiving alemtuzumab. In patients receiving alemtuzumab, serum levels of alemtuzumab were monitored to determine its persistence. The data showed detectable levels of alemtuzumab up to day 28. Results indicated that UCAR19 failed to proliferate in the patients not receiving alemtuzumab. However, expansion of UCAR19 was noted in 15 out of the 17 patients receiving alemtuzumab but persisted only up to day 28 in 86% of the patients. The recovery of endogenous T cells and the lack of UCAR19 persistence in the absence of alemtuzumab point to the development of a host T cell-mediated immune response leading to the clearance of UCAR19.

## Strategies to Overcome the Immunogenicity-Related Risk of CAR-T Cells

**Figure 2:**
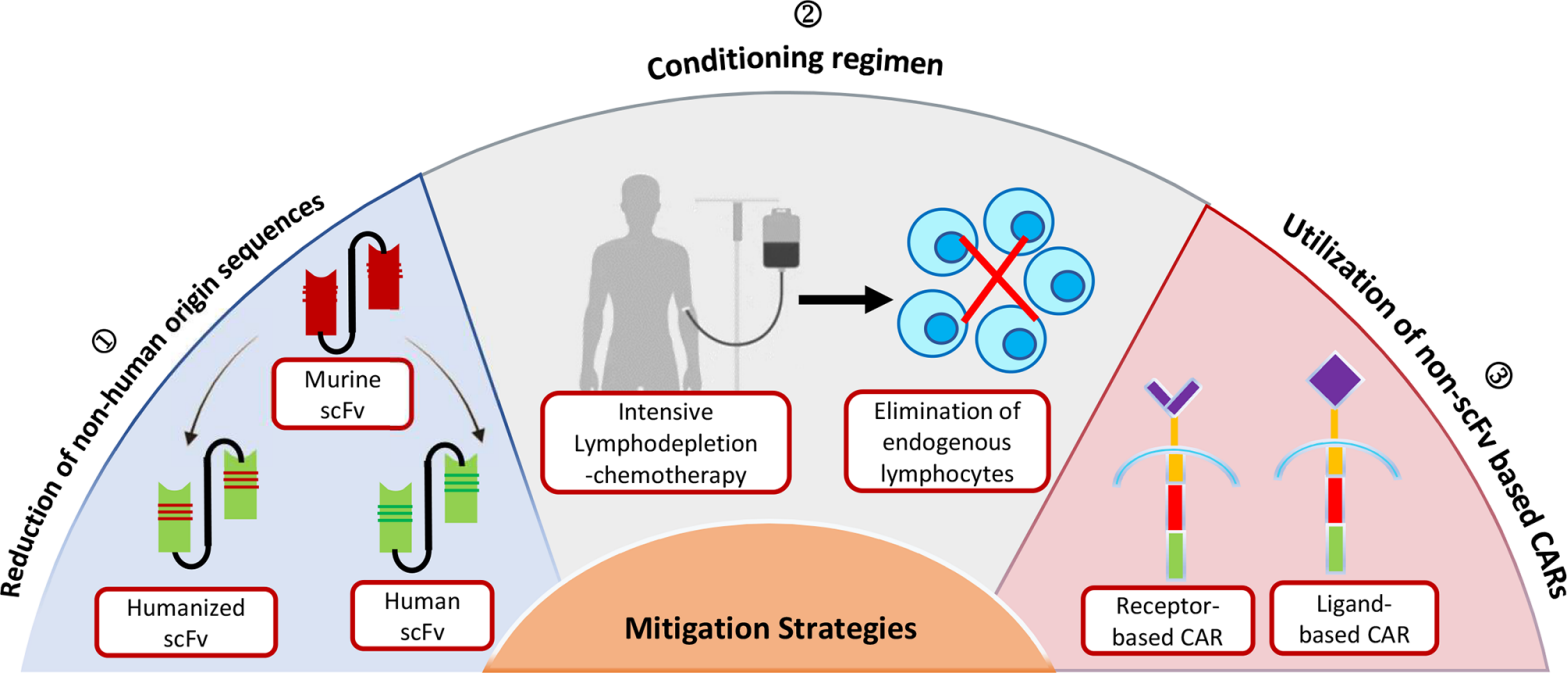
Strategies for limiting the Immunogenicity of Chimeric Antigen Receptor (CAR) T cell.

Based on the evidence presented above, non-human scFvs can be extrapolated as the primary cause of immunogenicity induction. Immune responses to the murine antigen-binding domain of CAR-T cells have been interconnected to CAR-T cell early clearance, rendering them ineffective and increasing the risk of tumor relapse. As a result, the following strategies could serve as a possible solution to limit the anti-CAR immune response while potentially improving the treatment benefit **(** **Figure 2****)**.

### Replacing Murine scFv With Fully Human Binding Domain

To overcome the risks posed by having protein sequences of non-human origin, recent CAR studies have implemented a complete switch from scFvs originating from murine to scFvs containing fully human binding sequences (75). Clinically, CAR-T cells with scFv derived from fully-human monoclonal antibodies (HuCAR-T) have also shown considerable promise, with reduced immunogenicity and toxicity, greater persistence, and a better clinical outcome (76).

In 2020, Jennifer N. Brudno and team underlined the clinical importance of integrating human binding domains in CAR design. Murine scFv of FMC63-28ξ construct employed in the previous study was substituted by a fully human anti-CD19 antibody-derived scFv (Hu19scFv) in a first-in-human phase I clinical trial (n=20, NCT02659943). Upon assessment of immunogenicity, a substantial reduction in anti-CAR immune response was detected in patients treated with CAR-T cells containing Hu19scFv (Hu19CAR) when compared to FMC63-28ξ treated patients. Furthermore, replacing scFv as such had no consequence on Hu19CAR function. Despite the overall alteration in the binding moiety, Hu19CAR demonstrated anti-tumor activity without inducing any notable side effects in any of the patients (77).

With an aim to overcome the immune rejection of CAR-T cells with murine binding domains, Sommermeyer et al., generated CD19-specific CAR with fully human scFv. Preliminary computational analysis was performed using the NetMHC algorithm, which revealed CAR sequences that were not encoded by the human genome. Further screening based on MHC binding prediction revealed that the fusion site between CD28 and 4-1BB contained seven 9-mer peptide sequences with <100nm affinity to several MHC class I molecules. To reduce the associated-risk, the CD28 sequence was extended by two amino acids, which resulted in only one predicted 9-mer peptide with an affinity of <100nm. Despite the modifications, human CD19CAR remained effective and safe when compared to FMC63-based CAR in both *in vitro* and *in vivo* model (78). This study suggests that using fully human CARs as a clinical target and removing potential immunogenic sequences from the CAR construct can effectively reduce immunogenic responses and also enable multiple repeat infusions while preserving its efficacy.

In a separate study, Lam et al., designed an anti-BCMA CAR (FHVH33-CD8BBξ) with a fully human heavy-chain variable domain (FHVH) to circumvent anti-CAR immune responses in r/r multiple myeloma as discussed above. The FHVH domain was derived from antibodies produced by specific transgenic rats (UniRats) post BCMA-immunization. UniRats have been shown to express heavy chain-only antibodies (HCAbs) derived from large transgenic loci that represents the entire functional human heavy chain (VDJ) repertoire. CARs with such antigen-recognition domains can help eliminate the risk of anti-CAR immunogenicity associated with murine-based CARs. In this study, FHVH33-CD8BBξ demonstrated robust efficacy both *in vitro* and *in vivo*, with no functional disadvantage when compared to murine anti-BCMA CAR (11D5-3). Findings of this study provide the foundation for designing CARs with lower immunogenicity by using only human heavy chain domains rather than murine sequences (59). In a separate study, CT053, a high affinity BCMA-specific CAR containing a fully human single chain fragment variant (25C2), was tested for efficacy and safety in patients with r/r multiple myeloma (n=24). During the phase I (LUMMICAR-1) trial (NCT03975907), an overall response rate (ORR) of 87.5% and a complete remission (CR) of 79.2% were achieved without immunogenicity induction in any subjects (79).

Similarly, a few other studies have highlighted the significance of human antigen recognition domain in CAR design in order to overcome the immunological barriers caused by CARs that are not entirely human in origin (80). Overall, fully human scFvs can minimize immunogenicity while also extending the longevity of CAR-T cells and improving therapeutic output in patients. Repeat dosing and dose escalation are also possible with such a strategy (59, 77). Further improvement in the use of human scFvs can be achieved by validating the aforementioned in other cancer targets.

### Humanization of Murine scFv

Murine scFvs in CAR design have shown a number of drawbacks, including immune rejection, reduced persistence, and treatment-related toxicities (81). Under such circumstances, humanizing murine sequences could be a feasible alternative. In this process, murine CDR sequences are grafted onto human framework region, thus reducing the foreignness in CAR design without loss of its binding properties (82). Humanizing murine scFvs could help reduce immunogenicity. This strategy can also resolve other toxicity-related issues, improving the efficacy of adoptive cellular therapies (83).

A number of humanized CAR-T cells have been developed and tested in preclinical and clinical settings, and they have shown promise in improving CAR immunogenicity, efficacy, and persistence by exhibiting variable binding affinity and cytokine release while retaining comparable anti-tumor activity and persistence. In 2010, Harding A. Fiona and team showed that replacing variable (V) region of the murine framework of cetuximab with the humanized equivalent significantly reduces the CD4+ T cell activation indicating the potential of humanization reduces immunogenicity of monoclonal antibody (84). Another study, led by Noreen R. Gonzales and colleagues, used patient sera treated with murine and humanized CC49 antibodies to perform an *in vitro* immune reactivity assay. The humanized Ab was developed by engrafting murine CD49 CDRs into the variable light (VL) and variable heavy (VH) frameworks of the human Abs LEN and 21/28’CL, respectively. However, some murine framework residues that were required for antigen binding to carcinomas were retained. The study analysis revealed that humanized CC49 antibody had lower reactivity than murine CC49 antibody (85). In another study, they illustrated that replacing some crucial amino acid residues in the murine framework region with their human counterparts further reduces the reactivity of humanized CC49 Ab towards anti-huCC49 Ab while maintaining comparable binding affinity, implying reduced immunogenicity (86).

Similarly, in multiple studies (NCT02349698, NCT02374333) 60-80% of patients treated with humanized CD19 CAR-T cells (huCAR-T19) showed complete remission for a longer time, ranging from 10-18 months, and detectable CAR copy number, indicating higher persistence and survival with humanized CAR-T cells. Another clinical evidence suggests the huCAR-T19 showed superior durable response, where 74 patients of r/r B-ALL treated with huCAR-T19 showed 24 months relapse free survival along with 6 months improved CAR-T cell persistence compared to murine CD19 CAR-T cells. In addition, when compared to patients treated with the murine CD19 CAR-T cell, there was a 6.5% reduction in Grade 4 CRS and neurotoxicity (87–90). These evidence suggest that humanization can aid in improving therapeutic cell persistence and mitigating the risks associated with the inclusion of non-human sequences.

We recently reported the development of two humanized CD19CAR (h1CAR19 and h2CAR19) with humanization in the framework region (FR) of FMC63-derived scFv as well as their role in improving the efficacy to toxicity balance. This was accomplished by grafting CDR residues into the framework of heavy chain,VH4-34, and light chain, VK1-O18. Furthermore, certain amino acid residues located near the CDRs and known to play a critical role in antigen binding were identified using PyMol software and Vernier zone identification by Kabat numbering. S^25^, I^69^, K^70^, and F^78^ were identified as the four key murine residues which were then conserved in the humanized FR. Although the two humanized scFvs had identical FR, their CDRs differed. h1CAR19 contains CDRs of a previously published humanized anti-CD19 CAR whereas CDRs for h2CAR19 were obtained from FMC63 mAb (91). As a result of these changes, the binding affinity and flexibility of CARs were altered. According to the Molecular dynamics (MD) simulation analysis, the scFv of h1CAR19 exhibited a higher binding affinity as well as flexibility upon binding to CD19 antigen. Findings from *in vitro* and *ex vivo* studies revealed that h1CAR19 secreted significantly lower IFN-ϒ and IL-2 while retaining its anti-tumor potential when compared to the other two CARs (mCAR19 and h2CAR19). Moreover, mice treated with h1CAR19 survived longer than mice treated with mCAR19, suggesting that the two CARs differ in their binding affinity and functional responses (92). Such improvements in h1CAR19 persistence imply that selecting appropriate sequences and modifying them through humanization are critical to reduce immunogenicity and other toxicities while preserving its efficacy. The study also hypothesizes that h1CAR19 may potentially reduce the risk of immunogenicity in clinical settings where multiple dosing is required.

In a separate study, Zhao et al., demonstrated the superiority of humanized selective CAR (hs CAR) in r/r B-ALL patients whose disease progressed after receiving murine anti-CD19 CAR-T cells (ChiCTR1800014761 and ChiCTR1800017439). 5 patients who had relapsed following murine-based CAR-T cell therapies were then treated with hsCD19-specific CAR-T cells. 2 of the patients previously received mCAR-T treatments twice but the repeat dose proved to be ineffective. In contrast, subsequent hsCAR-T treatments were effective in all 5 patients, with 4 achieving complete remission. Additional testing revealed that the 3 patients treated with mCAR were seropositive for IgA reactive to the extracellular domain of murine CD19CAR, whereas the patients treated with hsCAR-T cells had no such immune reaction (33). This study sheds light on the potential use of humanized CAR for the treatment of patients who have relapsed after receiving murine CAR-T cell therapies. Such studies, if conducted on large cohorts, will be able to better explain whether humanized CARs can assist in overcoming immunogenicity-related problems.

The said strategy, however, has not always resulted in the complete elimination of immune reactions against the CAR. Because of the residual murine sequences, humanized CAR retains the potential to elicit immune responses (93). According to Hege et al., patients treated with humanized anti-CD49 CAR-T for colorectal cancer expressing TAG-72 had an anti-CAR immune response, resulting in early clearance of therapeutic cells from the patients circulation (94). As a result, multiple clinical studies are required to confirm conclusively whether humanization of scFv can prove nonimmunogenic and reduce other toxicities-associated challenges.

### Pre-Infusion Treatment Regimen in Mitigating Immunogenicity

The success of CAR-T cell therapy is closely tied with pre-infusion immunosuppression by lymphodepletion. Lymphodepletion refers to administration of one or more chemotherapeutic agents which eliminates the patient lymphocytes. The role of lymphodepletion has been widely established in improving outcome of CAR-T cell therapy. Ramos et. al., conducted a phase I study of CD30.CAR-T cells infused in patients with r/r CD30^+^ HL or NHL with, or without preceding chemotherapy (95). The data showed improved expansion of CAR-T cells after lymphodepletion, which could be correlated to enhanced anti-tumor efficacy - 6/8 CR with lymphodepletion versus 1/6 CR without lymphodepletion. The patients in the study were administered Cy or Cy and etopside (E) for lymphodepletion before CAR-T cell infusion. To determine whether lymphodepletion had any effect on the prevention of anti-CAR immune responses, subsequent patients were administered Cy and Flu for lymphodepletion. On intensifying lymphodepletion, persistence of CAR-T cells up to several months was noted, as well as improved efficacy as compared to the patients receiving Cy or Cy/E. Further, 3 out of 4 patients who received Cy/Flu before the first CAR-T infusion and were given a second dose of CAR-T cell reported CAR-T cell expansion and tumor regression after the second dose without development of anti-CAR immune response unlike the response in the 5 patients not receiving Flu. The study therefore highlighted the importance of the lymphodepletion regime in managing immunogenic responses, leading to a better treatment outcome.

In a Phase I/II trial of P-BCMA-101 CAR-T cells in r/r multiple myeloma patients, novel therapeutic strategies were examined (NCT03288493). For this study, a group of patients received with rituximab or lenalidomide pre- and post-lymphodepletion, in addition to the standard 3-day lymphodepletion strategy (Cy/Flu). As a result, a 100% ORR with minimal side effects was reported. Such strategies, if used both before and after lymphodepletion, could help to prevent the generation of antibodies against CAR-T cells while also increasing CAR-T cell robustness (96).

An independent study led by Gauthier et al., additionally highlighted the importance of lymphodepletion techniques in relation to the clinical result after the second infusion of CD19-targeted therapy (NCT01865617). Before the CAR-T cell infusions, patients in the study either did not receive lymphodepletion (n=10) or were given a variable amount of Cy/Flu (n=34). A chromium release assay was used to evaluate anti-CAR immune responses to the CAR-transgene. According to the findings of this study, 50% of patients who did not receive lymphodepletion exhibited anti-CAR responses, whereas only 17% of patients who obtained Cy/Flu developed anti-CAR responses. After the second infusion, this impact was seen to enhance even more. Immunogenic responses were identified in 100% of Non-Cy/Flu patients versus 44% of Cy/Flu patients. Furthermore, the results revealed a connection between lymphodepletion and clinical outcome. In contrast to patients who did not receive Cy/Flu, patients who received Cy/Flu had a higher remission rate. Improved CAR-T cell expansion and persistence was observed after incorporating lymphodepletion strategy before first (CAR-T1) and second (CAR-T2) infusion. Additionally, endogenous CD8^+^ T-cell mediated immune responses against CAR-T cells were observed in a significant number of non-Cy/Flu patients (50% in post-CAR-T1, 88% post-CAR-T2) when compared to Cy/Flu treated patients (17% post-CAR-T1, 20% post-CAR-T2) (97). This implies that intensifying lymphodepletion prior to the first dose can minimize immune rejection while also improve *in vivo* CAR-T cell kinetics. If necessary, such a strategy might allow for a higher CAR-T cell repeat dose. Overall, this research indicates that lymphodepletion could be acting to mitigate anti-CAR immune responses, allowing patients to have more durable responses after repeat infusions, thus ultimately enhancing the overall clinical outcomes.

### Utilization of Non-scFv Based CARs

Induction of anti-CAR immunogenic response has been credited to the scFv moiety. An upcoming area of development is to generate CAR-T cells which utilize antigen binding domains other than the classical scFvs for tumor antigen recognition. Such CAR-T cells express human origin adaptors enabling them to interact with tumor cells with reduced immunogenicity. Receptor ligand CARs express a naturally occurring ligand/receptor on its surface, with affinity for cell surface markers found on tumor cells. Immune response is likely to be limited against the chimera incorporating a natural ligand or receptor-based model and prevent the clearance of the CAR-T cells. NKG2D CAR is a receptor-based CAR which targets stress-associated ligands expressed on many cancer types showing promise against multiple indications including leukemia, lymphoma, ovarian cancer, gastric cancer and Ewings’ sarcoma (98). Ligand based CARs expressing cytokines or a cytokine-derived receptor-binding peptides, hormones, and growth factors are also under development for the treatment of multiple solid cancers. Ongoing trials are in line with the heregulin (α/β)-ζ mediated CAR-T where heregulin serves as the antigen recognition domain, targeting Her3/Her4 antigen overexpressed on the tumor (99). The novel chimeric molecule designed provides a well-established instance of anti-tumor response generated in an MHC-independent fashion leading to eviction of the tumor population while maintaining T cell persistence. Apart from reduced immunogenicity, receptor ligand CARs allow non-tumor restricted therapeutic activity, targeting multiple tumor types which express the corresponding ligand/receptor. **Table 1**. summarizes the different receptor-ligand CAR-T cell strategies in development, with reduced immunogenic potential.

**Table 1:** Receptor-ligand CAR-T cell strategies in development.

CAR ectodomain	CAR-T cell	Indications	Advantages	Drawbacks	Trial status
Naturally occurring receptors	NKG2D CARs (100, 101)	Multiple myeloma, Acute myeloid leukemia, myelodysplastic syndrome, ovarian cancer, breast cancer, colorectal cancer, pancreatic cancer, bladder cancer	Non-immunogenic Variety of tumor associated ligands found on multiple tumor types	Higher risk of off target response Immune evasion by shedding of NKG2DL on tumors APRIL based CARs showed poor clinical efficacy	NCT04167696 NCT03466320 NCT03018405 NCT03692429
	TrCD27 CARs (102)	pancreatic cancer, renal cell cancer, breast cancer, ovarian cancer			NCT02830724
Cytokine or a cytokine-derived receptor-binding peptide	IL-11Rα-specific CAR T cells (103)	Metastatic osteosarcoma			Preclinical
	IL-13Rα2 CAR T cells (104, 105),	IL-13Rα2 expressing glioblastoma			NCT02208362
	CD116L CARs (106),	Juvenile myelomonocytic leukemia			Preclinical
	APRIL based CARs (107, 108),	Multiple myeloma			NCT03287804
Hormones	Follicle Stimulating Hormone (FSH) CAR (109).	Ovarian Cancer			Preclinical
Growth factors	T1E CAR (110).	ErbB1/ErbB2+3 expressing Breast cancer Head and neck squamous cell carcinoma Ovarian cancer			NCT01818323
	Heregulin (α/β) CAR-T cells (99).	HER3+ breast cancer Ovarian cancer, Prostate cancer and Gastric cancer			Preclinical

Another strategy that has evolved to prevent CAR-T cell clearance due to immune rejection is decoupling of the scFv moiety from the CAR-T cell. The CAR-T cells express human origin linkers or adaptors which interact with tumor antigen specific scFvs or antibodies. Universal CARs have been developed allowing a single CAR-T cell to target multiple tumors using different adaptor molecules. SUPRA CAR was developed using human transcription factor derived zipper components which allowed CAR-T cells to target multiple target antigens with reduced immunogenicity (111). The SNAP CARs encode a modified O-6-methylguanine-DNA methyltransferase enzyme (SNAPtag) at the extracellular side of a second-generation CAR, allowing it to bind with tumor antigen specific Fab or mAb which have been tagged with benzylguanine (112). The human origin enzyme component reduces chances of immune response induction.

Both the above CAR-T technologies disconnect the immunogenic murine scFv from the CAR-T cell thus reducing the impact of any immune response on the therapeutic cell. Furthermore, this strategy avoids constitutive expression of the antigen-binding moiety, rather allowing for transient administration of the scFv or antibody. Even in the event of clearance of the murine scFv, the CAR-T cells remain in circulation and can be reactivated by administration of the antigen binding molecule.

## Conclusion and Future Prospective

CAR-T cell therapy represents an advanced biotherapeutic modality in the field of cancer immunotherapy. Despite the fact that CAR-T cells have demonstrated robust anti-tumor potential in a range of malignancies, the therapy’s benefits are limited by the risks associated with it. The sparse understanding of therapy-associated risks, such as the potential of CAR-T cells to elicit a humoral and/or cellular immune response, adds to the complexity of developing effective CAR-Ts. This review highlights the studies that have investigated into and encountered such responses, their impact on clinical outcomes, along with strategies that could alleviate immunogenicity.

The significance and extent of immunogenicity induction has been primarily linked to the presence of non-human sequences in the CAR construct. As previously discussed, the majority of cases where immunological responses against CAR-T cells were reported employed scFv’s derived from murine origin. Antibodies developed against murine-derived scFvs were also shown to be more common in patients with relapse after CAR-T cell therapy. Furthermore, CAR-specific cytolytic-T cells have also been shown to be associated with immunological rejection of CAR-Ts, resulting in disease progression. Immunogenicity against CAR-T cells also led to resistance to repeat dosing, limiting the therapeutic index of CAR-T cells. However, the clinical relevance of anti-CAR immune responses is still not clearly understood. Anti-CAR antibodies against axicabtagene ciloleucel and tisagenlecleucel were reported in a set of patients during the trial study, however; these generated antibodies had no effect on the overall clinical outcome (51). On a similar note, Lee et al., reported induction of cellular immunity, but this immune rejection could not be related to the treatment outcome, as several patients with T cell-mediated anti-CAR immunity demonstrated sustained anti-leukemic affects (57). Approaches such as humanization of scFv or utilizing complete human binding domains can be considered as a potential strategy to reduce immunogenicity against adoptive transfer therapy. Secondly, an appropriate lymphodepletion strategy prior to infusion could assist to minimize anti-CAR immune responses, ultimately improving the overall outcome of CAR-T cell therapy. The use of non-scFv based CARs might additionally serve to mitigate immunogenic reactions.

Despite the various mitigation strategies/approaches available, identification and implementation of appropriate strategies to manage anti-CAR responses remains an unsolved problem. While multiple studies have shown that intensifying lymphodepletion can reduce anti-CAR responses, the phase I ZUMA trial also reported a lack of immune response induction but at a lower dose of conditioning treatment (38, 97, 113).

This suggests there still exists a lacuna in the field, and that little is understood about the correlation between immunogenicity and clinical outcome. Such disparities necessitate vigilance regarding potential CAR-related immunogenicity as well as a thorough understanding of the immunological response following CAR-T cell infusion. Appropriate monitoring and improvements in clinical management are required to ensure that CAR-T cell elimination is reduced, thereby minimizing the risks associated with infusion. A detailed analysis in both preclinical and clinical investigations is required to comment on the impact of immunogenicity on the ultimate outcome of the therapy and its implications. These data will help to fill the gap in our understanding of the clinical relevance of immunogenicity, paving the way for the development of more effective and safer future therapies.

## Author Contributions

RP supervised, conceptualized and edited the article. AN and AC conceptualized, and wrote the article. AK, AJ, AB, and SA made substantial contributions to writing and review of the article. All authors contributed to the article and approved the submitted version.

## Funding

The work was financially supported by the Indian Council of Medical Research (RD/0121-ICMR000-001) and Biotechnology Industrial Research Assistance Council (RD/0120-BIRAC01-002) at IIT Bombay to RP.

## Conflict of Interest

The authors declare that the research was conducted in the absence of any commercial or financial relationships that could be construed as a potential conflict of interest.

## Publisher’s Note

All claims expressed in this article are solely those of the authors and do not necessarily represent those of their affiliated organizations, or those of the publisher, the editors and the reviewers. Any product that may be evaluated in this article, or claim that may be made by its manufacturer, is not guaranteed or endorsed by the publisher.
